# Abundances of microRNAs in human cells can be estimated as a function of the abundances of YRHB and RHHK tetranucleotides in these microRNAs as an ill-posed inverse problem solution

**DOI:** 10.3389/fgene.2013.00122

**Published:** 2013-07-01

**Authors:** Mikhail P. Ponomarenko, Valentin V. Suslov, Petr M. Ponomarenko, Konstantin V. Gunbin, Irina L. Stepanenko, Oleg V. Vishnevsky, Nikolay A. Kolchanov

**Affiliations:** ^1^Department of Systems Biology, Institute of Cytology and Genetics SB RASNovosibirsk, Russia; ^2^Department of Natural Sciences, Novosibirsk State UniversityNovosibirsk, Russia; ^3^Academic Council, National Research Centre “Kurchatov Institute”Moscow, Russia

**Keywords:** microRNA, *Argonote*, miRNA/Ago-affinity, miRNA abundance, quantitative sequence-activity relationship (QSAR), ill-posed inverse problem, linear-additive approximation, “limiting stage” approximation

## Abstract

Mature microRNAs (miRNAs) are small endogenous non-coding RNAs 18–25 nt in length. They program the RNA Induced Silencing Complex (RISC) to make it inhibit either messenger RNAs or promoter DNAs. We have found that the mean abundance of miRNAs in Arabidopsis is correlated with the abundance of DRYD tetranucleotides near the 3′-end and the abundance of WRHB tetranucleotides in the center of the miRNA sequence. Based on this correlation, we have estimated miRNA abundances in seven organs of this plant, namely: inflorescences, stems, siliques, seedlings, roots, cauline, and rosette leaves. We have also found that the mean affinity of miRNAs for two proteins in the *Argonaute* family (Ago2 and Ago3) in man is correlated with the abundance of YRHB tetranucleotides near the 3′-end and that the preference of miRNAs for Ago2 is correlated with the abundance of RHHK tetranucleotides in the center of the miRNA sequence. This allowed us to obtain statistically significant estimates of miRNA abundances in human embryonic kidney cells, HEK293T. These findings in relation to two taxonomically distant entities (man and Arabidopsis) fit one another like pieces of a jigsaw puzzle, which allowed us to heuristically generalize them and state that the miRNA abundance in the human brain may be determined by the abundance of YRHB and RHHK tetranucleotides in these miRNAs.

## Introduction

MicroRNAs (miRNAs) are small endogenous non-coding RNAs (Kozomara and Griffiths-Jones, 2010). Within the canonical biogenesis of miRNAs, their genes are transcribed by RNA polymerase II into the primary transcripts (pri-miRNAs). Special Microprocessor proteins cut away the first precursor of miRNA (pre-miRNA) and then the mature miRNA 18–25 nt in length (Kozomara and Griffiths-Jones, 2010). The miRNAs that maturate from other sources, including spliced-out introns (they are the source of mirtrons) and transfer RNAs (tRNAs), are called “non-canonical.” They are less abundant in cells and their maturation time is deviant (Havens et al., 2012).

Mature miRNAs program the RISC (RNA-Induced Silencing Complex) to make it inhibit either messenger RNAs (mRNAs) or promoter DNAs (pDNAs) through the formation of mRNA(pDNA):miRNA-RISC complexes (Song et al., 2004). The function of the RISC depends upon what of the proteins in the *Argonaute* family is incorporated in the RISC (Gagnon and Corey, 2012). In the 3D structure of the mRNA:miRNA-Ago-RISC in Archaea (Song et al., 2004), the Ago protein interacts with the 3′-end of the miRNA.

Changes in mature miRNA abundance and sequence affecting interactions between the miRNAs and their targets were associated with various abnormalities, including neurodegeneration (Barbato et al., 2009) and cancer (Winter and Diederichs, 2011a). Winter and Diederichs (2011b) showed experimentally that the miRNA abundance in Ago2-deficient cells treated by the transcription inhibitor actinomycin D increases when the Ago2 protein is introduced to them ectopically, because the affinity of a particular miRNA for Ago2 protein influences the half-life of this miRNA in cells. Furthermore, Martinez and Gregory (2013) showed that Ago2 expression in mouse embryonic stem cells, originally low in Ago2 and then transfected by a vector containing Ago2, is dependent on miRNA abundance post-transcriptionally. Therefore, that miRNAs and human Ago2 stabilize each other is an experimentally established fact. How can we benefit from this fact?

Although nobody has measured *in vivo* the affinity of mature miRNAs for the different *Argonaute* proteins (Azuma-Mukai et al., 2008) or the abundance of these miRNAs in cells (Axtell and Bartel, 2005) under identical experimental conditions simultaneously, we have earlier demonstrated (Ponomarenko et al., 2001) that the patterns and features found *in silico* in one experiment readily apply to the next, at least within the limits of applicability of the theory that underlies these experiments. Consequently, we have had to work with disembodied experimental data on two taxonomically distant entities (man and Arabidopsis) using original ACTIVITY tools (Ponomarenko et al., 1997). As a result, we have successfully found correlations which fit each other like pieces of a puzzle created in two experiments, one by Winter and Diederichs (2011b) and another by Martinez and Gregory (2013), the mutual complementarity of which was, in fact, the starting point of our work. These correlations allowed us to generalize them into a heuristic hypothesis stating that miRNA abundance in the human brain depends on the abundance of YRHB and RHHK tetranucleotides in these miRNAs. This hypothesis was further confirmed using independent experimental data taken from the Sestan Brain Atlases (Kang et al., 2011).

The results obtained are discussed in terms of the “limiting stage” approximation, the linear-additive approximation, and an ill-posed inverse problem. This allowed us to conclude that *in silico* estimates like these can reach an acceptable accuracy level for their practical consideration by cancer and neurodegeneration researches once the preference of these miRNAs for the proteins in the *Argonaute* family has become known, and so have yet unknown values of the affinity of any miRNA for two of the four proteins (50%), Ago1 and Ago4, which is absolutely required for a more accurate approximation.

## Materials and methods

### Nucleotide sequences

The nucleotide sequences of the mature canonical Arabidopsis miRNAs {ξ_i_} were taken from a work by Axtell and Bartel (2005), ξ ∈ {a, u, g, c}. Seventeen out of 27 miRNAs were used as the training dataset (Table 1). Because miRNA lengths varied from 20 to 22 nt, our *in silico* processing was only confined to miRNAs of a given length (in Tables 1, 2, these sequences are typed in CAPITALS).

**Table 1:** **The training dataset (this work) of miRNA abundances in Arabidopsis (Axtell and Bartel, 2005) contains 17 miRNA fragments each 20 nt in length (they are in CAPITALS)**.

**Experimental data *in vivo* (Axtell and Bartel, 2005)**			**Analysis *in silico* (this work)**	
**miRNA**	**Canonical miRNA sequence**	**ln[miRNA], ln-unit**	**[WRHW]**_**F1**_	**[DRYD]**_**F2**_
ath-mir-156	UGACAGAAGAGAGUGAGCAC	3.09	0.97	2.08
ath-mir-157	UUGACAGAAGAUAGAGAGCAc	1.72	1.89	1.02
ath-mir-158	UCCCAAAUGUAGACAAAGCA	4.85	1.79	2.01
ath-mir-159	UUUGGAUUGAAGGGAGCUCUa	5.21	1.30	1.40
ath-mir-160	UGCCUGGCUCCCUGUAUGCCa	3.81	0.69	2.43
ath-mir-161.1	UGAAAGUGACUACAUCGGGGt	4.68	1.22	1.87
ath-mir-161.2	UCAAUGCAUUGAAAGUGACUa	3.90	1.78	1.63
ath-mir-163	UUGAAGAGGACUUGGAACUUcgau	1.96	0.61	1.70
ath-mir-164	UGGAGAAGCAGGGCACGUGCa	4.24	1.64	1.46
ath-mir-165	UCGGACCAGGCUUCAUCCCCc	0.90	0.00	0.70
ath-mir-166	UCGGACCAGGCUUCAUUCCCc	1.48	0.00	0.70
ath-mir-168	UCGCUUGGUGCAGGUCGGGAa	4.02	1.00	1.25
ath-mir-169	CAGCCAAGGAUGACUUGCCGa	2.11	0.00	1.61
ath-mir-171	UGAUUGAGCCGCGCCAAUAUc	2.02	0.48	1.17
ath-mir-390	AAGCUCAGGAGGGAUAGCGCc	2.65	0.43	2.13
ath-mir-394	UUGGCAUUCUGUCCACCUCC	2.00	0.00	0.25
ath-mir-398	UGUGUUCUCAGGUCACCCCUg	1.74	0.57	0.35
Coefficient of linear correlation			*r* = 0.67	*r* = 0.58
Statistical significance			α < 0.005	α < 0.025

**Table 2:** **The training dataset (this work) of miRNA affinities for the human Ago2 and Ago3 proteins (Azuma-Mukai et al., 2008); 12 miRNA fragments each 22 nt in length are in CAPITALS**.

**Experimental data *in vivo* (Azuma-Mukai et al., 2008)**				**Analysis *in silico* (this work)**			
**miRNA**	**Canonical miRNA sequence**	**[miR/Ago2] ln-un., X**_**2**_	**[miR/Ago3] ln-un., X**_**3**_	**Δ, (X_2_−X_3_)/2**	**[RHHK]_F3_**	**Σ, (X_2_+X_3_)/2**	**[YRHB]_F4_**
hsa-mir-342	*u*CUCACACAGAAAUCGCACCCGU	7.81	4.85	1.48	1.95	6.33	0.77
hsa-mir-21	UAGCUUAUCAGACUGAUGUUGA	8.34	7.24	0.55	1.59	7.79	1.45
hsa-mir-378	ACUGGACUUGGAGUCAGAAGGC	4.97	5.89	−0.46	1.26	5.43	0.00
hsa-mir-629	GUUCUCCCAACGUAAGCCCAGC	5.28	7.98	−1.35	0.91	6.63	0.37
hsa-mir-92b	UAUUGCACUCGUCCCGGCCUCC	6.48	8.98	−1.25	0.22	7.73	1.06
hsa-mir-221	AGCUACAUUGUCUGCUGGGUUU	6.08	5.26	0.41	2.37	5.67	0.50
hsa-mir-29c	UAGCACCAUUUGAAAUCGGUUA	6.08	6.05	0.02	1.36	6.06	0.42
hsa-mir-210	CUGUGCGUGUGACAGCGGCUGA	6.81	7.96	−0.57	0.59	7.38	1.32
hsa-mir-let7d	CUAUACGACCUGCUGCCUUUCU	6.49	6.46	0.01	1.53	6.47	1.00
hsa-mir-99b	CACCCGUAGAACCGACCUUGCG	8.55	6.70	0.92	1.43	7.62	2.09
hsa-mir-191	CAACGGAAUCCCAAAAGCAGCU	6.40	7.47	−0.53	1.05	6.94	0.82
hsa-mir-425	*a*AUGACACGAUCACUCCCGUUGA	7.33	8.50	−0.59	0.10	7.92	1.99
Coefficient of linear correlation				*r* = 0.75		*r* = 0.86	
Statistical significance				α < 0.005		α < 0.001	

The other 10 miRNAs (Figures 6, 7) were used as an independent experimental dataset (sequences not shown). Twenty-two Arabidopsis miRNAs taken from a work by Lu et al. (2005) were used as independent experimental control datasets (Figure 4C; sequences not shown).

The nucleotide sequences of human mature miRNA were taken from a work by Azuma-Mukai et al. (2008). Twelve out of 28 mature canonical miRNAs were used as the training dataset (Table 2).

The other 16 canonical miRNAs were used as independent experimental control datasets (Figure 3), and 48 miRNAs named the “individual variants” by Azuma-Mukai et al. (2008) because of their 5′- and/or 3′-terminal differences from canonical mature miRNAs, which were associated by Azuma-Mukai et al. (2008) with (i) alternative maturation (Azuma-Mukai et al., 2008) or (ii) post-maturation processing (Azuma-Mukai et al., 2008), 96 human miRNAs taken from a work by Bail et al. (2010), and 318 human miRNAs taken from miRBase (Kozomara and Griffiths-Jones, 2010) according to their identifiers in the Sestan Brain Atlases (Kang et al., 2011) (Figures 7, 8, 9, respectively; sequences not shown).

### Biological activity

The relative values ranging from −0.5 to 7.8 ln for the miRNA abundance in Arabidopsis taken from a work by Axtell and Bartel (2005) are partly presented in Table 1 and fully in Figures 3, 4 (the *y*-axis).

The relative values ranging from 0 to 4 ln for the miRNA abundance in Arabidopsis obtained using Massively Parallel Signature Sequencing (MPSS) were taken from a work by Lu et al. (2005) and used as an independent experimental control dataset (Figure 4, the *y*-axis).

The values ranging from 4.85 to 9.43 ln for the *in vivo* measured affinity of canonical miRNAs for the human Ago2 and Ago3 proteins were taken from a work by Azuma-Mukai et al. (2008), are partly presented in Table 2 and fully in Figures 5, 6 (the *y*-axis), while those for the affinity of 48 miRNAs named the “individual variants” by Azuma-Mukai et al. (2008) because of their 5′- and/or 3′-terminal differences from canonical mature miRNAs, which were associated by Azuma-Mukai et al. (2008) with (i) alternative maturation (Azuma-Mukai et al., 2008) or (ii) post-maturation processing (Azuma-Mukai et al., 2008), are shown in Figure 7.

The relative values ranging from −9.0 to 0.0 ln for the abundance of 96 human miRNAs in the human embryonic kidney cells HEK293T, some preincubated for 8 h with the transcription inhibitor actinomycin D and others not preincubated, were taken from a work by Bail et al. (2010) and used as an independent experimental control dataset (Figure 8, the *y*-axis).

The relative values ranging from 0 to 16 rel. un. for the miRNA abundance measured within 95 human brain regions or neocortical areas were taken from the Sestan Brain Atlases (Kang et al., 2011) and used as an independent experimental control dataset (Figure 9, the *y*-axis).

### Correlations between biological activity and miRNA nucleotide sequences

We have used our original development called ACTIVITY (Ponomarenko et al., 1997), which is a tool intended for the processing of input data on a pre-set biological activity, X({ξ_i_}) in known miRNA sequences, {ξ_i_} and searching for correlations in them.

Although ACTIVITY has been described in detail elsewhere (Ponomarenko et al., 1997), we will additionally provide a brief descriptions of its features that were critical to our current study.

**Figure 1:**
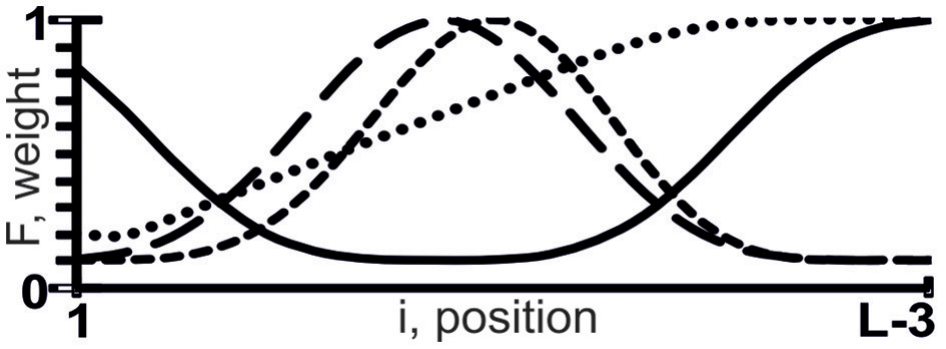
**Sample weights (the *y*-axis), F(i), of tetranucleotide z_1_z_2_z_3_z_4_ at the *i*-th position (the *x*-axis) of the sequence {ξ_i_} composed of L nt, with which we assessed the linear additive contribution to X({ξ_i_}) using the rule “the higher F(i), the greater the contribution**.”

First of all, ACTIVITY (Ponomarenko et al., 1997) searches for correlations between the biological activity of a miRNA, X({ξ_i_}), (expressed as its expression level; herein, as the experimentally measured abundance and miRNA/Ago affinity) and the weighted abundance of the tetranucleotides, [z_1_z_2_z_3_z_4_]_F_, in the sequence {ξ_i_} of this miRNA:

(1) $${\left[{\text{z}}_{1}{\text{z}}_{2}{\text{z}}_{3}{\text{z}}_{4}\right]}_{\text{F}}\left\{\xi_{1\leq \text{i}\leq \text{L}}\right\}=\sum_{\begin{array}{c} 1\leq \text{i}\leq \text{L−3}\\ \xi_{\text{i}}\xi_{\text{i}+1}\xi_{\text{i}+2}\xi_{\text{i}+3}\in {\text{z}}_{1}{\text{z}}_{2}{\text{z}}_{3}{\text{z}}_{4} \end{array}}\text{F}\left(\text{i}\right);$$

where z ∈ {a, u, g, c, w, r, m, k, y, s, b, v, h, d, n} (IUPAC-IUB, 1971); 0 ≤ F(i) ≤ 1 is the weight of the tetranucleotide z_1_z_2_z_3_z_4_ at the i-th position, with which we heuristically assessed its linear additive contribution to the X({ξ_i_}) value using the rule “the higher F(i), the greater the contribution” (Figure 1).

ACTIVITY (Ponomarenko et al., 1997) has built-in F(i) profiles: 180 U-shaped and 180 S-shaped curves for F(i) values, in which low and high weights have different locations and interval lengths. ACTIVITY works uniformly on each of all the possible variants of weighted tetranucleotide abundance ([z_1_z_2_z_3_z_4_]_F_), their number being 360 × 15^4^ = 18225000 ≈ 10^7^.

Furthermore, each [z_1_z_2_z_3_z_4_]_F_{ξ_i_} value was compared with X({ξ_i_}) using bootstrapping (Hayes et al., 1989) in 7 subsets: (i) the entire dataset; (ii) 50% of the entries that have the lowest [z_1_z_2_z_3_z_4_]_F_{ξ_i_} values; (iii) 50% of entries that have the highest [z_1_z_2_z_3_z_4_]_F_{ξ_i_} values; (iv) 50% of the entries that are closest to the mean of all the [z_1_z_2_z_3_z_4_]_F_{ξ_i_} values; (v) 50% of the entries that have the lowest X({ξ_i_}) values; (vi) 50% of the entries that have the highest X({ξ_i_}) values; (vii) 50% of the entries that are closest to the mean of all the X({ξ_i_}) values. By varying statistical test data [bootstrapping (Hayes et al., 1989)], we seek to minimize the dependence of search results on the input dataset.

In each of these seven subsets, ACTIVITY (Ponomarenko et al., 1997) checks five types of correlation between X({ξ_i_}) and [z_1_z_2_z_3_z_4_]_F_({ξ_i_}): i) linear correlation; ii) Spearman's rank correlation; iii) Kendall's rank correlation; iv) dichotomous correlations tested by χ^2^; and v) dichotomous correlations tested by the Fisher-Irwin test. Because it is possible to obtain quantitative estimates using linear correlations, such correlations could be useful if it were not for their sensitivity to data heterogeneity. By contrast, dichotomous correlations do not depend on data heterogeneity; however, they provide the least informative estimates above/below any pre-set threshold. Based on the usefulness-to-robustness ratio, rank correlations are between linear and dichotomous correlations. We search the input training dataset for different types of correlation and identify the best trade-offs.

Furthermore, ACTIVITY (Ponomarenko et al., 1997) checks the following six criteria of the applicability of regression analysis to the {[z_1_z_2_z_3_z_4_]_F_({ξ_i_}); X({ξ_i_})} data: i) how uniform the X({ξ_i_}) values are; ii) how uniform the [z_1_z_2_z_3_z_4_]_F_({ξ_i_}) values are; iii) whether the departures of the [z_1_z_2_z_3_z_4_]_F_({ξ_i_}) values from the {λ X({ξ_i_})+μ} regression are normal; iv) whether the departures of [z_1_z_2_z_3_z_4_]_F_({ξ_i_}) from {λX({ξ_i_}) + μ} independent of each other; v) whether the departures of the X({ξ_i_}) values from the {φ[z_1_z_2_z_3_z_4_]_F_({ξ_i_})+ψ} regression are normal; and vi) whether the departures of the X({ξ_i_}) values from {φ[z_1_z_2_z_3_z_4_]_F_({ξ_i_}) + ψ} independent of each other. We search the input training dataset for the correlations that satisfy additional criteria for their applicability to making *in silico* estimates, for example, for estimating unknown X({ξ_i_}) values from known {ξ_i_} values based on the weighted abundance, [z_1_z_2_z_3_z_4_]_F_({ξ_i_}).

**Figure 2:**
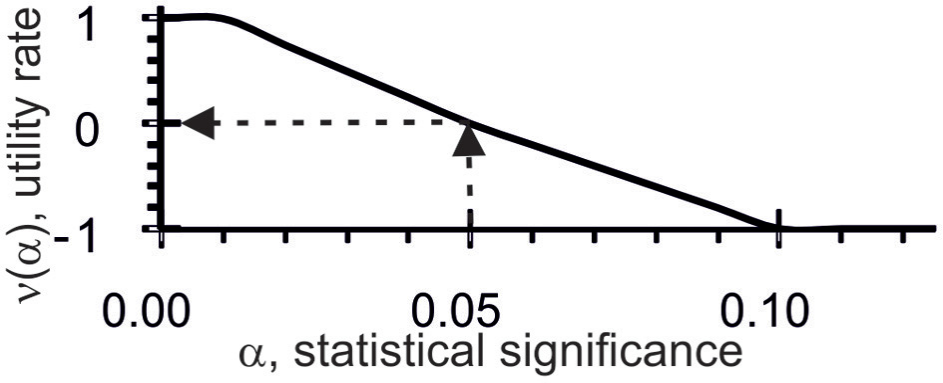
**Utility estimates (the *y*-axis), υ(α), for correlations between X({ξ_i_}) and [z_1_z_2_z_3_z_4_]_F_({ξ_i_}) based on statistical significance (α, the *x*-axis) for the given dataset**.

Thus, ACTIVITY (Ponomarenko et al., 1997) checks 11 criteria (five types of correlation and six criteria of the applicability of regressions) on each of 10^7^ [z_1_z_2_z_3_z_4_]_F_ variants and in each of 7 subsets yields 11 × 7 = 77 values for the statistical significance (α). In terms of fuzzy set theory (Zadeh, 1965) and utility theory for decision making (Fishburn, 1970), each α is transformed into a numerical utility value, α → υ(α), for the correlation between X({ξ_i_}) and [z_1_z_2_z_3_z_4_]_F_({ξ_i_}), see Figure 2.

As can be seen, the threshold α set at 0.05 was the reference point for υ = 0: when the tests were statistically significant, the utility values were positive and when the tests were not statistically significant, the utility values were negative. Each [z_1_z_2_z_3_z_4_]_F_ estimate in the input training dataset, X({ξ_i_}) and {ξ_i_}, was equal to their mean:

(2) $$\Xi ({\left[{\text{z}}_{1}{\text{z}}_{2}{\text{z}}_{3}{\text{z}}_{4}\right]}_{\text{F}}\left\{\xi_{\text{i}}\right\};\text{X}\left\{\xi_{\text{i}}\right\})=\frac{1}{77}\sum_{\text{k=1}}^{7}\sum_{\text{q=1}}^{11}\left(\alpha_{\text{kq}}\right).$$

Finally, in the given input training dataset {{ξ_i_}; X({ξ_i_})}, ACTIVITY (Ponomarenko et al., 1997) finds the only [z_1_z_2_z_3_z_4_]_F_ value with the highest Ξ([z_1_z_2_z_3_z_4_]_F_({ξ_i_};X({ξ_i_})) > 0 or infers that the correlations found with the input training dataset are useless.

### Verification of the correlations found

Because ACTIVITY finds the only best correlation from among 10^7^ variants in any given input training dataset (Ponomarenko et al., 1997), verification is absolutely required.

First of all, the Bonferroni test yields p(Ξ > 0) < 10^−20^ (Omelyianchuk et al., 2011). This implies that it is quite unlikely that half of the 77 tests run can be satisfied simultaneously for random chance at α < 0.05.

Also, the training dataset, {ξ_i_} and X({ξ_i_}), is brought to the permutation test (Sohn et al., 2009): all the data are randomly rearranged {{ξ^#^_i_}; X({ξ^$^_i_})} and fed to ACTIVITY. The lack of the same [z_1_z_2_z_3_z_4_]_F_ value or its correlated [z^#^_1_z^$^_2_z^&^_3_z^*^_4_]_F_ value in 100 independent cycles is statistically significant (α < 0.05; binomial law).

**Figure 3:**
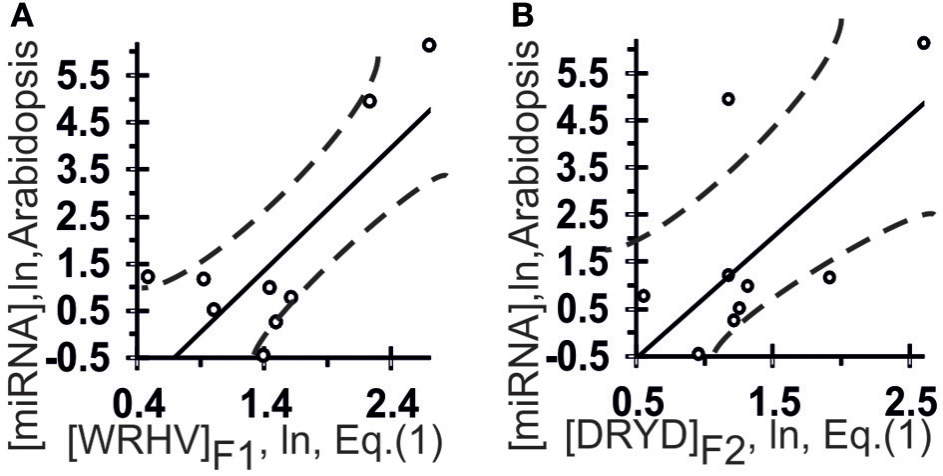
**Control test of the patterns (the *x*-axis) found by ACTIVITY (Ponomarenko et al., 1997) in the training dataset (Table 1) using independent experimental data (the *y*-axis) taken from the same data source (Axtell and Bartel, 2005)**. Two linear correlations in Arabidopsis: one **(A)** between miRNA abundance in the plant and [WRHB]_F3_ abundance in these miRNAs and one **(B)** between miRNA abundance and [DRYD]_F4_ abundance. Both are statistically significant in the control dataset of 11 miRNAs (Axtell and Bartel, 2005). Dashed curves depict 95% confidence intervals for linear regression (solid lines) built using STATISTICA (Afifi et al., 2003).

In turn, the statistical significance of the correlation found by ACTIVITY (Ponomarenko et al., 1997) with the training dataset is tested for on the control dataset with unprocessed experimental data (Figures 3, 5).

**Figure 4:**
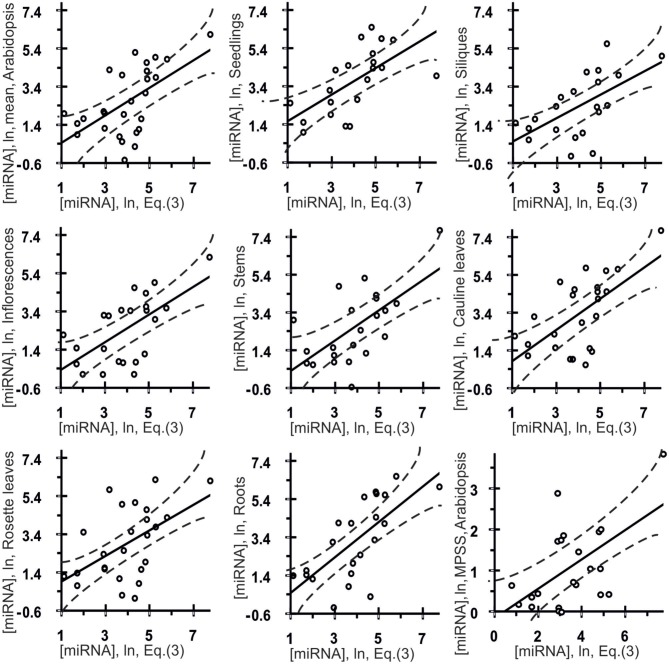
**Control test of the final equation (3), the *x*-axis, derived with ACTIVITY outputs (Ponomarenko et al., 1997) on the training dataset (Table 1) and using independent experimental data (the *y*-axis) taken from various data sources**. Twenty seven correlations in Arabidopsis (Table 3) between the miRNA abundance estimated by Equation (3), the *x*-axis, and those measured experimentally, the *y*-axis, namely: the mean abundances of 28 canonical miRNAs used above, the organ-specific abundance of these miRNAs in seedlings, siliques, inflorescences, stems, cauline leaves, rosette leaves, and roots as independently measured *in vivo* (Axtell and Bartel, 2005), and, finally, the abundances of 22 miRNAs obtained using Massively Parallel Signature Sequencing (MPSS) by Lu et al. (2005) as independent experimental control datasets. Dashed curves and solid lines as in the legend to Figure 3.

Finally, the statistical significance of the correlations found by ACTIVITY (Ponomarenko et al., 1997) with the given training dataset is tested using independent experimental data (Figures 4, 7–9).

### Cluster analysis of the miRNAs

In this work, we used standard statistical tools available in the STATISTICA system (Afifi et al., 2003), which has the “Joining (tree clustering)” mode in “Cluster” section under the “Multivariate/Exploratory” option in the “Statistics” part. Under this mode, we clustered all the RNAs being studied using all 42 = 7 × 6 possible combinations of seven Linkage rules: “Single linkage,” “Complete linkage,” “Unweighted pair-group average,” “Weighted pair-group average,” “Unweighted pair-group centroid,” “Weighted pair-group centroid,” and “Ward's methods,” and each from among six “Distance measures”: “Squared Euclidian distance,” “Euclidian distance,” “City-block (Manhattan) distance,” “Chebychev distance metric,” “Power,” “Percent disagreement,” and “1-Pearson r.” The color-coded results obtained from the most widely used (predefined) combination of the “Single linkage” rule and the “Euclidian distance” metric are shown in Figure 9. The results obtained from each of the other 41 combinations are not shown, because they have only minor deviations (less than 1% of RNAs) in the vicinity of the intercluster boundary caused by heterogeneity in experimental data.

## Results and discussion

### miRNA abundance in Arabidopsis

We ran ACTIVITY (Ponomarenko et al., 1997) on the experimental data (Axtell and Bartel, 2005) on the mean abundance of mature canonical ubiquitous miRNAs in Arabidopsis (Table 1, ln[miRNA]). We composed a training dataset (Table 1) for ACTIVITY (Ponomarenko et al., 1997) consisting of all the variants that had the lowest and highest values for miRNA abundance in seven organs of this plant (inflorescence, stem, silique, seedling, root, cauline and rosette leaves) and the occurrence of nucleotides A, U, G, C, W, R, and K in miRNAs (IUPAC-IUB, 1971). The resulting 17 out of 27 miRNAs in the training dataset (Table 1) represent the ranges of values of miRNA properties rather than data heterogeneity (Azuma-Mukai et al., 2008). The other 10 miRNAs were used as an independent experimental control dataset (Figure 3).

The highest estimated value, Ξ = 0.48, was assigned [Equation (2)] to the correlation between the abundance of miRNAs (ln[miRNA]) in Arabidopsis and the abundance of WRHW tetranucleotides ([WRHW]_F1_) with its highest weight, F1(i), in the center of the miRNA (Figure 1, short-dashed line). In the control dataset, the correlation between [WRHW]_F1_ and ln[miRNA] was statistically significant (Figure 3A: *r* = 0.74, α < 0.025).

Two more Ξ values were equal to 0.46 at the same WRHW tetranucleotide with narrower peaks (Ponomarenko et al., 2008). No other values Ξ > 0 were found in the training set. The next eight z_1_z_2_z_3_z_4_ tetranucleotides that had the highest Ξ-values were RYHV (Ξ_MAX_ = −0.01), RHWV (−0.05), DYDR (−0.07), SNKH (−0.07), RHWR (−0.08), SWBH (−0.09), BRHR (−0.11), and SVKH (−0.12) (in descending order).

For the S-shaped weights, the highest estimated value, Ξ = 0.47, was assigned [Equation (2)] to the abundance of the DRYD tetranucleotide ([DRYD]_F2_) with its highest weight, F2(i), at the 3′-end of the miRNA (Figure 1, dotted line). In the control dataset, the correlation between [DRYD]_F2_ and ln[miRNA] was statistically significant (Figure 3B: *r* = 0.66, a < 0.05). No other values Ξ > 0 were found in the training set (Ponomarenko et al., 2008). The next nine z_1_z_2_z_3_z_4_ tetranucleotides that had the highest Ξ-values were SNYW (Ξ_MAX_ = −0.02), WVVM (−0.04), RVYR (−0.05), VAHS (−0.06), VRDS (−0.07), RDMW (−0.08), DYDR (−0.09), RHWK (−0.18), and SVKH (−0.23) (in descending order).

Because [WRHW]_F1_ and [DRYD]_F2_ were independent (*r* = 0.39, α > 0.25), we skipped the optimization procedure and derived the following formula:

(3) $$\begin{array}{l} {}[\text{miRNA}]\left\{\xi_{\text{j}}\right\}=0.78+1.31{\left[\text{WRHW}\right]}_{\text{F1}}\left\{\xi_{\text{j}}\right\}\\ \;+0.76{\left[\text{DRYD}\right]}_{\text{F2}}\left\{\xi_{\text{j}}\right\}\text{​}. \end{array}$$

Estimates made with Equation (3) were statistically significantly (Figure 4, Table 3: *r* = 0.59, α < 0.0025) correlated with data reported by Axtell and Bartel (2005). As can be seen from Figure 4, the *in vivo* measured abundances of most miRNAs with calculated abundances ranging from 3.0 to 5.0 ln range from −0.5 to 5.5 ln (almost the full range of the graph). That is why the high *r*-value of the regression is probably due to the contribution of several anomalies like the most abundant miRNAs. To see if it is as it appears to be, we additionally estimated Spearmen's rank correlation coefficient (Table 3: *R* = 0.54; α < 0.005) and Kendall's rank correlation coefficient (Table 3: τ = 0.38; α < 0.01) (they consider the ranks of [miRNA] values rather than their true values) for *in silico* [miRNA] values (the *x*-axis) and *in vivo* [miRNA] values (the *y*-axis).

**Table 3:** **Twenty-seven correlations in Arabidopsis (Figure 4) between the miRNA abundance estimated by Equation (3) and those measured experimentally**.

**Experimental dataset**		**Linear correlation**		**Spearmen's rank correlation**		**Kendall's rank correlation**	
**No.**	**(Reference) organ**	**Coefficient *r***	**Significance α**	**Coefficient *R***	**Significance α**	**Coefficient τ**	**Significance α**
	Axtell and Bartel, 2005						
1	Means, plant	0.63	<0.001	0.54	<0.005	0.37	<0.01
2	Seedlings	0.63	<0.0025	0.69	<0.001	0.47	<0.005
3	Siliques	0.59	<0.005	0.60	<0.005	0.43	<0.01
4	Inflorescences	0.62	<0.0025	0.62	<0.0025	0.42	<0.005
5	Stems	0.64	<0.0025	0.59	<0.005	0.40	<0.01
6	Cauline leaves	0.63	<0.001	0.54	<0.005	0.37	<0.01
7	Rosette leaves	0.52	<0.01	0.51	<0.001	0.35	<0.025
8	Roots	0.69	<0.0005	0.73	<0.00025	0.55	<0.0005
	Lu et al., 2005						
9	Whole plant, MPSS	0.56	<0.01	0.46	<0.05	0.30	<0.05

MPSS, Massively Parallel Signature Sequencing.

Also, Figure 4 shows seven statistically significant linear correlations between the estimates obtained using Equation (3) and the abundances of miRNA in seven organs of Arabidopsis (Axtell and Bartel, 2005), namely: inflorescences, stems, siliques, seedlings, roots, cauline leaves and rosette leaves. The dashed curves in this figure depict the boundaries of the 95% confidence interval for the mean miRNA abundance in Arabidopsis estimated by Equation (3). As can be seen, despite the statistical significance in the correlations between the value defined by Equation (3) and miRNA abundance, a large part of data points in Figure 4 exist outside of the dashed lines. This implies that Equation (3) is an adequate source of rough estimates of miRNA abundances in Arabidopsis organs; however, there is a high variability of their organ-specific values (the coefficient of variation, C_V_ = σ/M_0_ × 100%, expressed as the percentage of the ratio between the standard deviation and the mean, ranging from 7 to 72%, the mean being 31 ± 19%) which was ignored by Equation (3) due to lack of data.

Finally, the three above mentioned correlations between *in silico* and *in vivo* [miRNA] values were statistically significant in the independent experimental dataset (Lu et al., 2005) [Table 3: *r* = 0.56 (α < 0.01), *R* = 0.46 (α < 0.05), τ = 0.30 (α < 0.05)]. Therefore, the statistical significance of 27 independent tests (Figure 4 and Table 3) is rather an argument for than against a dependence of miRNA abundance on tetranucleotide abundance in these miRNAs.

However, the molecular mechanism that Equation (3) is consistent with remains unclear. Admittedly, Hwang et al. (2007) established experimentally that the sequence of a mature miRNA is a factor for the efficiency of its export from the nucleus to the cytoplasm, and Gantier et al. (2011) explored effects of Dicer1 on the miRNA half-life in a context dependent manner (Gantier et al., 2011). If we were to consider Equation (3) together with the results of the experiments performed by Winter and Diederichs (2011b) and by Martinez and Gregory (2013) suggesting that miRNAs and Ago2 are likely to stabilize each other, it could be admitted that Equation (3) implies miRNA/Ago2 affinity.

### miRNA/Ago affinity in man

We ran ACTIVITY (Ponomarenko et al., 1997) simultaneously on two libraries (Table 2) of mature human miRNAs specific for either Ago2 or Ago3 (Azuma-Mukai et al., 2008). To this end, instead of using the affinity magnitude [miRNA/Ago2] and [miRNA/Ago3], we heuristically constructed two auxiliary estimates:

(4) $$\begin{array}{l} \Sigma =\left(\left[\text{miRNA/Ago2}]+[\text{miRNA/Ago3}\right]\right)/2;\\ \Delta =\left(\left[\text{miRNA/Ago2}]-[\text{miRNA/Ago3}\right]\right)/2. \end{array}$$

We composed a training dataset (Table 2) for ACTIVITY (Ponomarenko et al., 1997) consisting of all the variants that had the lowest and highest values for Σ, Δ, the abundance of nucleotides A, U, G, C, W, R, and K in miRNAs (IUPAC-IUB, 1971). The resulting 12 miRNAs in the training dataset (Table 2) represent the ranges of values of miRNA properties rather than data heterogeneity (Azuma-Mukai et al., 2008).

**Figure 5:**
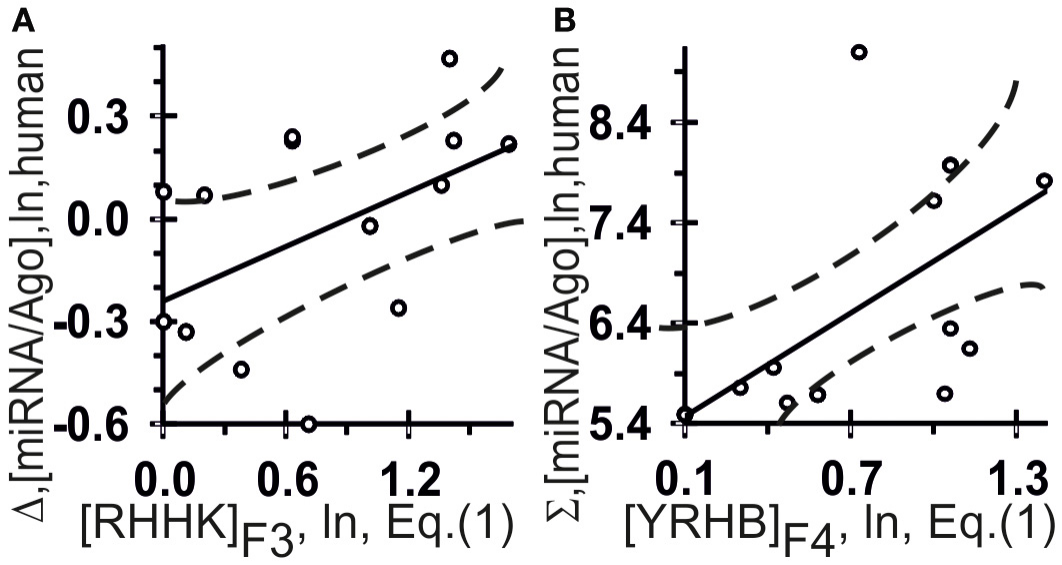
**Control test of the patterns (the *x*-axis) found by ACTIVITY (Ponomarenko et al., 1997) in the training dataset (Table 2) using independent experimental data (the *y*-axis) taken from same data source (Azuma-Mukai et al., 2008)**. Two statistically significant linear correlations: one **(A)** between the miRNA/Ago-affinity estimate (Δ) and [RHHK]_F3_; and one **(B)** between another estimate (Σ) and [YRHB]_F4_. Both are statistically significant in the control dataset of 16 canonical miRNAs (Azuma-Mukai et al., 2008). Dashed curves and solid lines as in the legend to Figure 3.

The highest estimated value, Ξ = 0.36, was assigned [Equation (2)] by ACTIVITY (Ponomarenko et al., 1997) to the correlation between Δ and the abundance, [RHHK]_F3_, of the RHHK tetranucleotide (IUPAC-IUB, 1971) with its highest weight, F3(i), in the center of the miRNA (Figure 1, broken line). This corresponds to the difference that Ago2 and Ago3 have in cleaving the mRNA in the center of its complementarity with the miRNA-Ago2(3)-RISC complex (Song et al., 2004). The [RHHK]_F1_ values for all the 12 miRNAs of the training dataset are presented in Table 2. The correlation between [RHHK]_F3_ and Δ was statistically significant (*r* = 0.75, a < 0.005), and so was that in the control dataset (Figure 5A: *r* = 0.51, a < 0.05). Another Ξ value, 0.34, indicated at the same tetranucleotide, RHHK, with a narrower peak, F(i), in the center of the miRNA. No other higher-than-zero Ξ values were found in the training dataset (Omelyianchuk et al., 2011). The next eight tetranucleotides that had the highest Ξ-values were WRHH (Ξ_MAX_ = −0.01), RBBM (−0.01), RDDK (−0.01), ABMD (−0.02), YWBM (−0.02), RBMD (−0.02), DSSV (−0.04), and WRMH (−0.06) (in descending order).

For Σ, the highest estimated value, Ξ = 0.36, was assigned [Equation (2)] to the abundance, [YRHB]_F4_, of the YRHB tetranucleotide (IUPAC-IUB, 1971) with its highest weight F4(i) at the 3′-end of the miRNA (Figure 1, solid line). This corresponds to the contact of the miRNA and the Ago protein in the 3D structure of the mRNA:miRNA-Ago-RISC complex (Song et al., 2004). The correlation between [YRHB]_F4_ and Σ was statistically significant in the control dataset (Figure 5B: *r* = 0.61, α < 0.025). No other values Ξ > 0 were found in the training set (Omelyianchuk et al., 2011). The next nine tetranucleotides that had the highest Ξ-values were RBMB (Ξ_MAX_ = (−0.01), ANKK (−0.01), DKSM (−0.12), MHKR (−0.13), YHKD (−0.14), HASH (−0.16), WNNS (−0.17), DDSM (−0.19), KMDK (−0.21) (in descending order).

**Figure 6:**
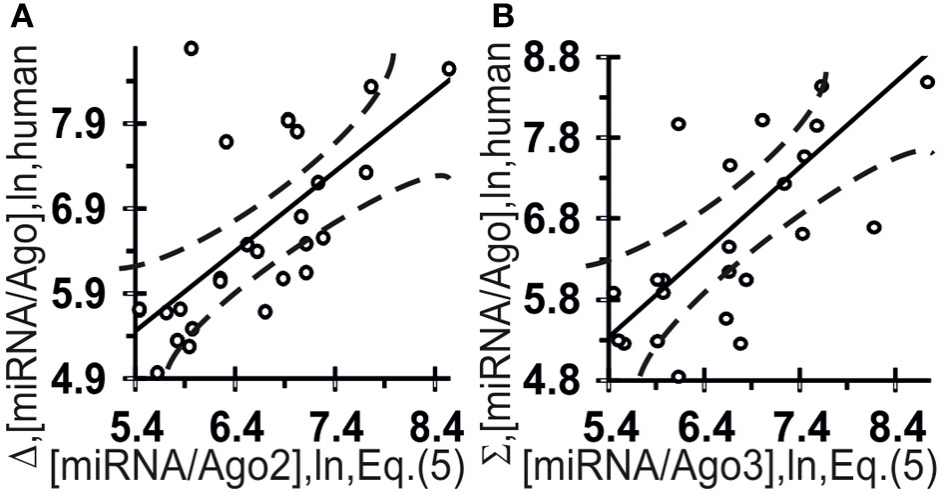
**Control test of the final Equation (5), the *x*-axis, derived with ACTIVITY outputs (Ponomarenko et al., 1997) on the training dataset (Table 2) and using independent experimental data (the *y*-axis) on the canonical miRNAs taken from the same data source (Azuma-Mukai et al., 2008)**. miRNA/Ago affinity as measured *in vivo* (Azuma-Mukai et al., 2008) and as estimated *in silico* and expressed in logarithms are statistically significantly correlated for Ago2 **(A)** and Ago3 **(B)**. Dashed curves and solid lines as in the legend to Figure 3.

Figure 6 shows independent estimates made on the basis of these two correlations for the affinity of miRNAs for Ago2 ([miRNA/Ago2] = Σ + Δ) and Ago3 ([miRNA/Ago3] = Σ − Δ) derived without optimization:

(5) $$\begin{array}{l} {}[\text{miRNA/Ago2}]\left\{\xi_{\text{j}}\right\}=4.97+0.52{\left[\text{RHHK}\right]}_{\text{F3}}\left\{\xi_{\text{j}}\right\}\\ \;+1.35{\left[\text{YRHB}\right]}_{\text{F4}}\left\{\xi_{\text{j}}\right\};\\ {}[\text{miRNA/Ago3}]\left\{\xi_{\text{j}}\right\}=6.11-0.52{\left[\text{RHHK}\right]}_{\text{F3}}\left\{\xi_{\text{j}}\right\}\\ \;+1.35{\left[\text{YRHB}\right]}_{\text{F4}}\left\{\xi_{\text{j}}\right\}. \end{array}$$

They are statistically significantly [Figure 6: (A) *r* = 0.66 and (B) *r* = 0.66, α < 0.00025] correlated with all the experimental data (Azuma-Mukai et al., 2008).

**Figure 7:**
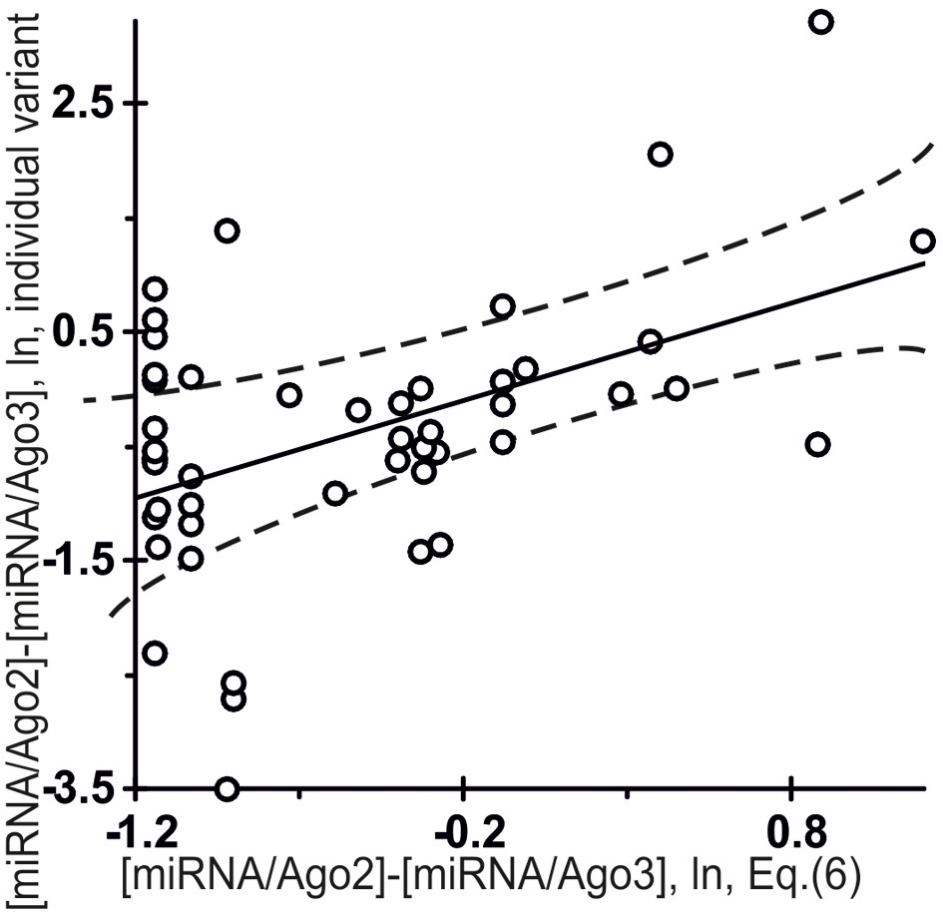
**Control test for the difference between the affinity of miRNAs for Ago2 and that for Ago3 (Equation (6), the *x*-axis) estimated using final Equation (5) and independent experimental data (the *y*-axis) on the miRNA individual variants taken from the same data source (Azuma-Mukai et al., 2008)**. The differential affinities of the Ago2 and Ago3 proteins for 48 miRNAs named the “individual variants” by Azuma-Mukai et al. (2008) because of their 5′- and/or 3′-terminal differences from canonical mature miRNAs, which were associated by Azuma-Mukai et al. (2008) with (i) alternative maturation (Azuma-Mukai et al., 2008) or (ii) post-maturation processing (Azuma-Mukai et al., 2008), as independently measured *in vivo* (Azuma-Mukai et al., 2008) and as estimated *in silico* [Equation (6)] and expressed in natural logarithms are statistically significantly correlated (Table 4). Dashed curves and solid lines as in the legend to Figure 3.

Figure 7 shows independent *in silico* estimates obtained using Equation (5) for 48 miRNAs named the “individual variants” by Azuma-Mukai et al. (2008) because of their 5′- and/or 3′-terminal differences from canonical mature miRNAs, which were associated by Azuma-Mukai et al. (2008) with (i) alternative maturation (Azuma-Mukai et al., 2008) or (ii) post-maturation processing (Azuma-Mukai et al., 2008). The estimated value was statistically significant (*r* = 0.49, α < 0.001) for the difference between the affinity of the miRNAs for Ago2 and that for Ago3:

(6) $$[\text{miRNA/Ago2}]-[\text{miRNA/Ago3}]=1.04{\left[\text{RHHK}\right]}_{\text{F3}}\left\{\xi_{\text{j}}\right\}-1.14.$$

This is consistent with the commonly accepted view that an individual miRNA variant forms complexes with Ago2 and Ago3 depending on its affinity for each of them, because specific interactions that normally occur due to evolutionary selection for affinity for these proteins are not there.

### An ill-posed inverse problem solution

**Figure 8:**
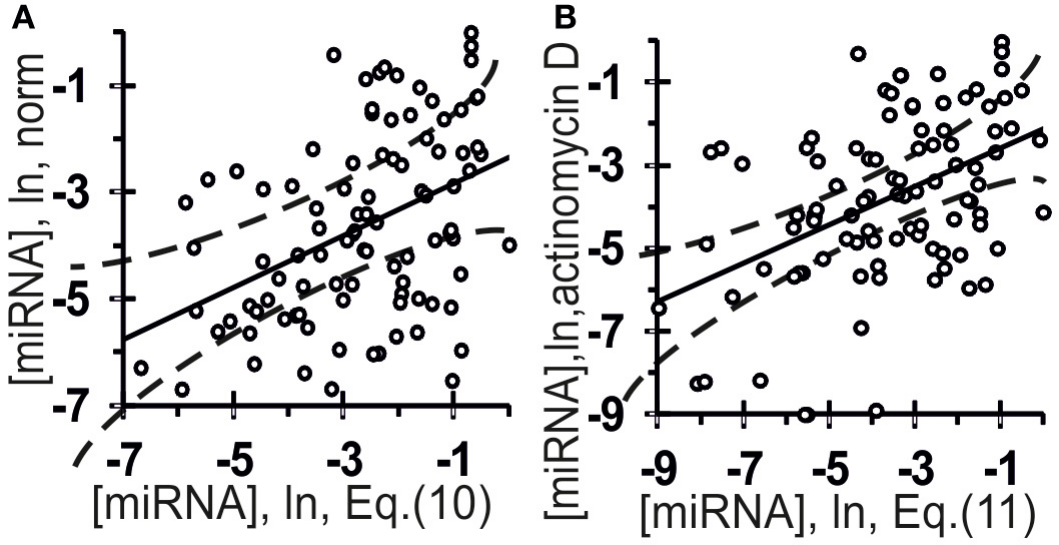
**Verification of ill-posed inverse problem solutions (Equation (10) and (11), the *x*-axis) using the final Equation (5) and independent experimental data (the *y*-axis) taken from another data source (Bail et al., 2010)**. The abundance of 96 mature miRNAs in an extract from the human embryonic kidney cell line HEK293T: **(A)** norm; **(B)** preincubation for 8 h with the transcription inhibitor actinomycin D (Bail et al., 2010). Independent experimental control data (*y*-axes) and *in silico* estimates and expressed on the same measurement scale [Equation (10) and Equation (11), respectively; *x*-axes] are statistically significantly correlated (Table 4). Dashed curves and solid lines as in the legend to Figure 3.

The values of the abundances of 96 mature miRNAs in an extract from human embryonic kidney cells, HEK293T, under normal conditions (A) and following preincubation for 8 h with the transcription inhibitor actinomycin D (Bail et al., 2010) (B) are on the *y*-axis in Figure 8. Let us see whether these values can be predicted using Equation (5) with miRNA nucleotide sequences known from the miRBase database (Kozomara and Griffiths-Jones, 2010).

On the one hand, under the normal experimental conditions in (Bail et al., 2010), a total amount of a certain miRNA was measured so that the experimental value [miRNA] should be described by the linear-additive approximation as follows:

(7) $$\begin{array}{l} {\left[\text{miRNA}\right]}^{\left(\#\right)}\left\{\xi_{\text{j}}\right\}=\beta_{1}^{\left(\#\right)}\left(\left[\text{Ago1}\right]\right){\left[\text{miRNA/Ago1}\right]}^{\left(\#\right)}\left\{\xi_{\text{j}}\right\}\\ \;+\beta_{2}^{\left(\#\right)}\left(\left[\text{Ago2}\right]\right){\left[\text{miRNA/Ago2}\right]}^{\left(\#\right)}\left\{\xi_{\text{j}}\right\}\\ \;+\beta_{3}^{\left(\#\right)}\left(\left[\text{Ago3}\right]\right){\left[\text{miRNA/Ago3}\right]}^{\left(\#\right)}\left\{\xi_{\text{j}}\right\}\\ \;+\beta_{4}^{\left(\#\right)}\left(\left[\text{Ago4}\right]\right){\left[\text{miRNA/Ago4}\right]}^{\left(\#\right)}\left\{\xi_{\text{j}}\right\}+\varepsilon ; \end{array}$$

where: β^(#)^_1_, β^(#)^_2_, β^(#)^_3_, and β^(#)^_4_ represent occupancies of the corresponding Ago1, Ago2, Ago3, and Ago4 proteins given an equilibrium of the miRNA and Ago molecule turnover in normal (#) HEK293T cells; ε is the prediction error, which inevitably creeps in due insufficient experimental data.

On the other hand, only two variables, [miRNA/Ago2]^(#)^ and [miRNA/Ago3]^(#)^, out of 8 can be estimated by Equation (5) if the experimental conditions (&) are different (Azuma-Mukai et al., 2008), [miRNA/Ago2]^(&)^ and [miRNA/Ago3]^(&)^. In addition, Ago1 and Ago2, but not Ago3, are the major Ago proteins in human, and the expression of Ago3 and Ago4 is low (Valdmanis et al., 2012). Therefore, it seems quite difficult, or probably impossible, to estimate the total miRNA amount from [RHHK]_F3_{ξ_j_} and [YRHB]_F4_{ξ_j_} since too many ambiguities exist and too small contribution of [miRNA/Ago3] to the total amount of the miRNA is logically expected for Equation (7). In this sense, Equation (7) is an “ill-posed inverse problem.”

We have recently proposed a solution to an ill-posed inverse problem (Mironova et al., 2013) using STATISTICA (Afifi et al., 2003) and considering the existing additional information given in the frames. In our case, this additional information is represented by two results of the experiments performed by Winter and Diederichs (2011b) and by Martinez and Gregory (2013) suggesting that miRNAs and Ago2 are likely to stabilize each other and that Ago2 is one of two major Ago proteins in human (Valdmanis et al., 2012). This representation substantiates the use of STATISTICA (Afifi et al., 2003) as a means of assessing the statistical significance of the linear-additive contribution of [miRNA/Ago2]^(#)^ estimates using Equation (5) in the linear-additive approximation by Equation (7) for experimental values [miRNA]^(#)^ as follows:

(8) $${\left[\text{miRNA}\right]}^{\left(\#\right)}\left\{\xi_{\text{j}}\right\}=\gamma^{\left(\&)\to (\#\right)}{\left[\text{miRNA/Ago2}\right]}^{\left(\&)\to (\#\right)}\left\{\xi_{\text{j}}\right\}+\delta^{\left(\&)\to (\#\right)};$$

where: γ^(&)→(#)^ and δ^(&)→(#)^ are the numerical values of the coefficients of dimension (constriction/compression and shifting, respectively) required for setting up a correspondence between the ranges of experimental variables found by Azuma-Mukai et al. (2008), (&), and by Bail et al. (2010) for normal HEK293T cells (#) without any optimization; [miRNA/Ago2]^(&)→(#)^{ξ_j_} is a heuristic estimate of an unknown [miRNA/Ago2]^(#)^{ξ_j_} value using the [miRNA/Ago2]^(&)^{ξ_j_} estimate and the final Equation (5).

The following formula was used as a heuristic estimate of [miRNA/Ago2]^(&)→(#)^{ξ_j_}:

(9) $$\begin{array}{l} {\left[\text{miRNA/Ago2}\right]}^{\left(\&)\to (\#\right)}\left\{\xi_{\text{j}}\right\}={\left[\text{miRNA/Ago2}\right]}^{\left(\&\right)}\left\{\xi_{\text{j}}\right\}/3\\ \;-{\left[\text{miRNA/Ago3}\right]}^{\left(\&\right)}\left\{\xi_{\text{j}}\right\}\text{​}; \end{array}$$

where: 1/3 is the heuristic coefficient that takes into account the normalization of experimental measurements (Azuma-Mukai et al., 2008) for Ago2 only in miRNA/Ago2 complexes within RISC without reference to Ago2 involvement in the regulation of transcription initiation or miRNA biogenesis;—[miRNA/Ago3]^(&)^{ξ_j_} is a heuristic correction, which takes into account a negative effect of the competition between Ago2 and Ago3 for miRNA binding and reduces [miRNA/Ago2]^(&)→(#)^{ξ_j_} in the measurements taken without Ago3 (&).

After all intermediate calculations, the final Equation (7) assumed the following form:

(10) $$\begin{array}{l} {}[\text{miRNA}]\left\{\xi_{\text{j}}\right\}=-2.74+1.32{\left[\text{RHHK}\right]}_{\text{F3}}\left\{\xi_{\text{j}}\right\}\\ \;-1.71{\left[\text{YRHB}\right]}_{\text{F4}}\left\{\xi_{\text{j}}\right\}; \end{array}$$

where: −2.74, 1.32, and −1.71 are the numerical values of the regression coefficients in Equation (7) via Equation (5).

The estimates obtained using Equation (10) were statistically significantly correlated with the measured [miRNA] values in normal HEK293T cells (Figure 8A, Table 4: *r* = 0.42, α < 0.000025; *R* = 0.43, α < 0.000025; and τ = 0.30 at α < 0.000025).

**Table 4:** **Nine correlations in human (Figures 7, 8) between the miRNA abundance estimated by Equation (5) and those measured experimentally**.

**Experimental dataset**	**Linear correlation**		**Spearmen's rank correlation**		**Kendall's rank correlation**	
**(Reference) conditions**	**Coefficient *r***	**Significance α**	**Coefficient *R***	**Significance α**	**Coefficient τ**	**Significance α**
Azuma-Mukai et al., 2008						
Individual variants	0.49	<0.001	0.30	<0.05	0.22	<0.05
Bail et al., 2010						
HEK293T, norm	0.42	<0.000025	0.43	<0.00005	0.30	<0.00025
HEK293T, actinomycin D	0.46	<0.000005	0.43	<0.00005	0.30	<0.00025

Nevertheless, there is an absolutely required additional stage in addressing an ill-posed inverse problem (Mironova et al., 2013), namely, verification using independent experimental data. To include this stage, we additionally reproduced all the calculations for experimental data under conditions that included ($) preincubation of HEK293T cells for 8 h with the transcription inhibitor actinomycin D (Bail et al., 2010). Because actinomycin D inhibits transcription elongation, the main difference between these conditions ($) and the normal conditions (#) is that no primary pri-miRNA transcripts are present and, consequently, Ago2-mediated miRNA biogenesis does not go. That is why we used 1/2 instead of 1/3 in Equation (9). After all intermediate calculations, the final Equation (10) derived from Equation (5) assumed the following form:

(11) $$\begin{array}{l} {}[\text{miRNA}]\left\{\xi_{\text{j}}\right\}=-4.56+2.20{\left[\text{RHHK}\right]}_{\text{F3}}\left\{\xi_{\text{j}}\right\}\\ \;-1.90{\left[\text{YRHB}\right]}_{\text{F4}}\left\{\xi_{\text{j}}\right\}. \end{array}$$

The estimates obtained using Equation (11) were statistically significantly correlated with the measured [miRNA] values at the HEK293T cells preincubated for 8 h with the transcription inhibitor actinomycin D (Bail et al., 2010), as shown in Figure 8B and Table 4: *r* = 0.46, α < 0.000005; *R* = 0.43, α < 0.000025; and τ = 0.30 at α < 0.000025). Nevertheless, despite the statistical significance in the correlations between the value defined by Equation (10) and (11) and miRNA abundance, a large part of data points in Figure 8 exist outside of the dashed lines. This implies that Equations (10) and (11) are adequate sources of rough estimates of miRNA abundances in human embryonic kidney cells, HEK293T, under proper experimental conditions consistent with Ago2 protein affinity for miRNAs; however there must be the Ago1 protein, which is another major Ago protein in man (Valdmanis et al., 2012) and which was ignored by Equations (10) and (11) due to lack of experimental data [miRNA/Ago1].

Collectively, all these results imply that Equation (5) produces adequate estimates ([miRNA]) for miRNA abundance in the given human cell line with an account of the biochemical features of the method used for experimental measurements [Equations (10) and (11) as examples of an ill-posed inverse problem solution].

Thus, Equation (5) found *in silico* in one experiment (Azuma-Mukai et al., 2008) readily applies to the next (Bail et al., 2010), at least within the limits of applicability of the theory that underlies these experiments. We had previously demonstrated this possibility (Ponomarenko et al., 2001), and its value is that it allows previously found patterns to be used for planning conditions of future experiments (for example, see Savinkova et al., 2013).

### Our hypothesis on miRNA abundance in the human brain

Thus, all the different types of correlation shown in Figures 3–8 fit each other like pieces of a puzzle, which allowed us to heuristically generalize all of them and state that the miRNA abundance in the human brain regions or neocortical areas may be roughly described by the function of YRHB and RHHK abundances in these miRNAs for their practical consideration by cancer and neurodegeneration researchers.

Let us check this hypothesis.

### An independent control test

**Figure 9:**
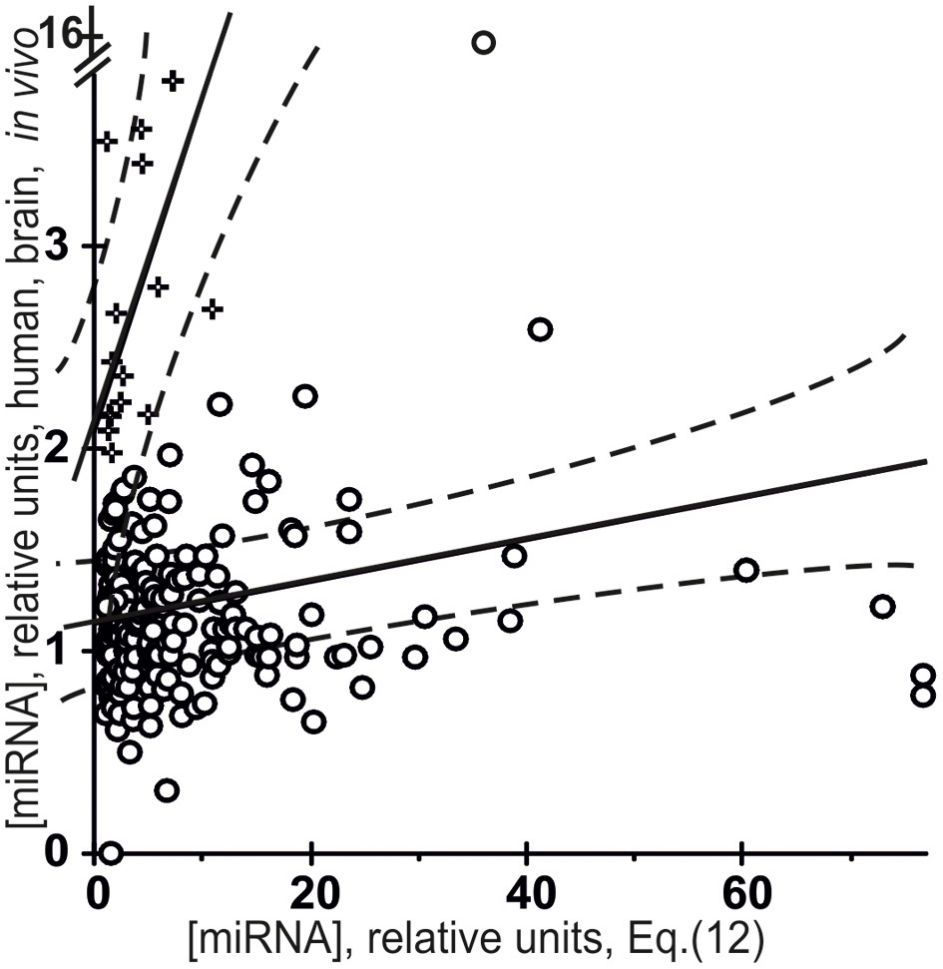
**Verification of the “limiting stage” approximation [Equation (12), the *x*-axis] using the final Equation (5) and independent experimental data (the *y*-axis) taken from the Sestan Brain Atlases (Kang et al., 2011)**. Results of a cluster analysis performed using STATISTICA (Afifi et al., 2003) on the miRNA abundance in the human brain taken from the Sestan Brain Atlases (Kang et al., 2011) vs. *in silico* estimates [Equation (12)] within the framework of the “limiting stage” approximation. The major cluster (○) includes 294 miRNAs (92%), which have the lowest mean miRNA abundance *in vivo*, [miRNA] = 1.2 ± 1.0, and a high mean [miRNA/Ago] affinity estimated [Equation (12)], 9.1 ± 12.7, and the lowest statistically significant correlation coefficient *r* = 0.14 (α < 0.025). The minor cluster (+) includes 24 miRNAs (8%) with the highest mean miRNA abundance *in vivo*, 2.7 ± 0.7, and a lowest mean estimate of [miRNA/Ago] affinity *in silico* [Equation (12)], 3.5 ± 2.8, and the highest correlation coefficient *r* = 0.66 (α < 0.001). Dashed curves and solid lines as in the legend to Figure 3.

Figure 9 presents the results of a cluster analysis performed using STATISTICA (Afifi et al., 2003) on the data on miRNA abundance in the human brain taken from the Sestan Brain Atlases (Kang et al., 2011), the *y*-axis, vs. *in silico* estimates obtained using [Equation (5)] within the framework of the roughest approximation possible, the so-called “limiting stage” approximation (the *x*-axis):

(12) $$\begin{array}{l} {}[\text{miRNA}]\left\{\xi_{\text{j}}\right\}=\text{exp}[\text{MIN}(0.52{\left[\text{RHHK}\right]}_{\text{F3}}\left\{\xi_{\text{j}}\right\}\\ \;+1.35{\left[\text{YRHB}\right]}_{\text{F4}}\left\{\xi_{\text{j}}\right\}\text{​};\\ \;1.14-0.52{\left[\text{RHHK}\right]}_{\text{F3}}\left\{\xi_{\text{j}}\right\}\\ \;+1.35{\left[\text{YRHB}\right]}_{\text{F4}}\left\{\xi_{\text{j}}\right\})]. \end{array}$$

It was applied due to lack of data on the preference of any of these miRNAs for any of the Ago1, Ago2, Ago3, or Ago4 protein in the *Argonaute* family (Gagnon and Corey, 2012) and also due to lack of data on the affinity of any miRNA for two of these four proteins (50%), Ago1 and Ago4. Moreover, not only cells specific for the central nervous system can influence the mean abundance of miRNAs in the human brain, but many more tissue-specific cells such as neurons, glia (microglia, oligodendrocytes, astrocytes, etc.), meninges (connective tissues covering the brain and containing a large number of blood vessels), cells of choroid plexus (capillaries, simple cuboidal epithelium, ependymal cells) and other can do as much. This diversity should increase the variance of [miRNA] values even more than in the case of the organ-specific abundance of miRNAs in Arabidopsis (Figure 4). Indeed, while the C_V_-values for miRNA abundance in Arabidopsis inflorescences, stems, siliques, seedlings, roots, cauline and rosette leaves ranged from 7 to 72% (the mean being 31 ± 19%), the C_V_-values for miRNA abundance in 95 human brain regions or neocortical areas ranged from 9 to 281% (the mean being 73 ± 39%), possibly due to very high levels of expression of unique miRNAs in a limited number of these regions or areas.

First of all, the major cluster (◦) includes 294 miRNAs (92%), which have a low mean miRNA abundance *in vivo*, [miRNA] = 1.2 ± 1.0, and a high mean [miRNA/Ago] affinity estimated *in silico* [Equation (12)], 9.1 ± 12.7. This cluster comprises miRNAs that have no preference for binding to any of the four proteins in the *Argonaute* family. This result is consistent with the statement used in the derivation of Equation (5) and made by Azuma-Mukai et al. (2008): most human miRNAs have no preference for binding to any particular Ago protein. We were surprised to see that even the roughest estimates were nevertheless statistically significantly linearly correlated (*r* = 0.14, α < 0.025) with *in vivo* measurements.

Finally, the minor cluster (+) includes 24 miRNAs (8%) with a high mean miRNA abundance *in vivo*, 2.7 ± 0.7, and a low mean estimate of [miRNA/Ago] affinity *in silico* [Equation (12)], 3.5 ± 2.8. This cluster contains miRNAs, each of which has a preference for binding to one particular Ago protein. This result is consistent with conclusions made by Azuma-Mukai et al. (2008): a few miRNAs in man have preference for binding to any particular Ago protein. As can be seen, these roughest estimates are, again, statistically significantly (*r* = 0.66, α < 0.001) correlated with *in vivo* values. Importantly, a higher *r*-value for specific than non-specific miRNA/Ago affinity (0.66 > 0.14) is in agreement with the most common view of the interactions between molecules. Nevertheless, despite the statistical significance in the correlations between the value defined by Equation (12) and miRNA abundance in the human brain, a large part of data points in Figure 9 exist outside of the dashed lines. Therefore, Equation (12) is an adequate source of only roughest estimates of miRNA abundances in the human brain; however, there is a wealth of relevant information on the Ago1 and Ago4 proteins (Figure 8), on the tissue-specific patterns of miRNA and Ago gene expression (Figure 4), which was ignored by Equation (12) due to lack of experimental data.

## Concluding remarks

We have now established that miRNA abundances depend on taxon-specific tetranucleotides in miRNAs.

First of all, specific tetranucleotides in a given miRNA seem to be responsible for the selectivity of miRNA binding to the proper Ago protein, which determines the biological function of the RISC containing this miRNA/Ago complex: (i) the RISC interacts with promoter DNAs or messenger RNAs (mRNAs) as it searches them for a complementary target of these miRNAs; and (ii) the RISC binds to or cleave this target within the mRNAs (Gagnon and Corey, 2012).

Based on these facts, we have for the first time obtained quantitative *in silico* estimates for miRNA abundances in the human embryonic kidney cells HEK293T by roughly solving an ill-posed inverse problem, and, also, in the human brain regions or neocortical areas, which are statistically significantly correlated with data from independent experiments on measuring these values *in vivo* taken from a work by Bail et al. (2010) and from the Sestan Brain Atlases (Kang et al., 2011), respectively. These two correlations are consistent with the results of two experiments, one performed by Winter and Diederichs (2011b) and another, by Martinez and Gregory (2013), and demonstrated that the affinity of miRNAs for Ago proteins is an influence on the abundance of both miRNAs and Ago proteins due to their mutual co-stabilization in cells.

In summary, we have found evidence that *in silico* estimates like these can reach an acceptable accuracy level for their practical consideration by cancer and neurodegeneration researches once the preference of these miRNAs for the proteins in the *Argonaute* family has become known, and so have yet unknown values of the affinity of any miRNA for two of the four proteins (50%), Ago1 and Ago4, which is absolutely required for a more accurate approximation. In any case, because the abundance estimates [Equations (5)—(12)] for most miRNAs were more statistically significant in a particular human cell line (Figure 8) than in the human brain as a whole (Figure 9), the more specifically a target for estimation is defined (the entire human organism, an organ, a part, a tissue, a cell type, or a cell line), the more suitable these estimates are for practical use.

### Conflict of interest statement

The authors declare that the research was conducted in the absence of any commercial or financial relationships that could be construed as a potential conflict of interest.
